# The mediating effect of the cyberchondria and anxiety sensitivity in the association between problematic internet use, metacognition beliefs, and fear of COVID-19 among Iranian online population

**DOI:** 10.1016/j.heliyon.2020.e05135

**Published:** 2020-10-10

**Authors:** Seyed Ghasem Seyed Hashemi, Shalaleh Hosseinnezhad, Solmaz Dini, Mark D. Griffiths, Chung-Ying Lin, Amir H. Pakpour

**Affiliations:** a Department of Psychology, Faculty of Psychology and Educational Sciences, Azarbaijan Shahid Madani University, Tabriz, Iran; b Department of Psychology, Urmia Branch, Islamic Azad University, Urmia, Iran; c Department of Psychology, Faculty of Psychology and Educational Sciences, Bonab Branch, Payame Noor University, Bonab, Iran; d International Gaming Research Unit, Psychology Department, Nottingham Trent University, Nottingham, UK; e Department of Rehabilitation Sciences, Hong Kong Polytechnic University, Hung Hom, Hong Kong; f Institute of Allied Health Sciences, National Cheng Kung University Hospital, College of Medicine, National Cheng Kung University, Tainan, Taiwan; g Social Determinants of Health Research Center, Research Institute for Prevention of Non-Communicable Diseases, Qazvin University of Medical Sciences, Qazvin, Iran; h Department of Nursing, School of Health and Welfare, Jönköping University, Jönköping, Sweden

**Keywords:** Psychology, COVID-19, Cyberchondria, Internet, Metacognition, Psychological distress

## Abstract

With the rapid growth of the novel coronavirus disease 2019 (COVID-19), individuals may try to find related medical information using the internet to overcome their fears. Under such circumstances, individuals with the features of cyberchondria, anxiety sensitivity, and metacognitive beliefs in negative thoughts may suffer more fears than those without these features. Therefore, the present study proposed a model to understand the associations between problematic internet use (PIU), cyberchondria, anxiety sensitivity, metacognition beliefs, and fear of COVID-19. Utilizing a cross-sectional online survey, 651 Iranians completed the following psychometric scales: Metacognition Questionnaire-30 (MCQ-30), Anxiety Sensitivity Questionnaire (ASI), Cyberchondria Severity Scale-Short Form (CSS-12), Fear of COVID-19 Scale (FCV–19S), and Generalized Problematic Internet Use Scale (GPIUS). Structural equation modeling (SEM) was used to assess the proposed model via several fit indices. The indices include Tucker-Lewis index (TLI), comparative fit index (CFI), standardized root mean square residual (SRMR), and root mean square error of approximation (RMSEA). The fit indices (CFI = 0.948, TLI = 0.938, RMSEA = 0.053, and SRMR = 0.001) indicated the good fit between the data and the proposed model. Moreover, fear of COVID-19 was significantly and directly predicted by cyberchondria (β = 0.479, *p* < .001) and anxiety sensitivity (β = 0.286, *p* < .001). The relationship between PIU and cyberchondria with fear of COVID-19 was mediated significantly by anxiety sensitivity and metacognitive beliefs. Because fear of COVID-19 was found to be significantly associated with cyberchondria and anxiety sensitivity, healthcare providers may want to provide additional support for those with cyberchondria and anxiety sensitivity tendencies.

## 1 Introduction

The novel coronavirus disease 2019 (COVID-19), which has plagued the world in recent months, has been the cause of more than 950,000 deaths around the world at the time of writing [1]. The virus has had devastating effects on human social life in many countries and in some cases has had serious consequence including death [2, 3, 4]. High-speed contamination and relatively high mortality have raised concerns about COVID-19, and many individuals have reported a fear of COVID-19 [5]. Many recent studies have investigated psychological issues related to COVID-19 [6, 7, 8, 9, 10, 11, 12]. Among the current literature on COVID-19 and psychological health, some psychosocial factors have been found to be crucial in causing and increasing COVID-19 fears that result in serious health problems [5, 10, 13, 14].

The internet is a resource that individuals use to address their concerns about various aspects of their lives [7, 15]. Information and medical issues are also widely available on the internet in most countries worldwide [16, 17]. Indeed, evidence shows that more than 50% of internet users search for and study medical information through online news, newspapers, and magazines [17, 18]. Studies have also shown that the use of the internet and social networking sites for stress and anxiety reduction has increased during the COVID-19 pandemic, and for some individuals, this stress and anxiety reduction may be accompanied by problematic internet use (PIU) [15, 19, 20]. Searching for medical information on the internet can also be a problem if individuals have little or no medical education, especially when web searches are used as a diagnostic method because this can increase their anxiety [16].

The latest (eleventh) revision of the International Classification of Diseases (ICD-11) notes that individuals with hypochondriac illness have a strong desire to seek medical information because of fear [21]. When individuals with hypochondriasis characteristics use the internet and social media networks to search for medical information, they are conceptualized as having cyberchondria [22]. Cyberchondria has not been identified as a separate diagnosis in the latest (fifth) edition of the Diagnostic and Statistical Manual of Mental Disorders (DSM-5) [23], but it is a type of anxiety disorder in which individuals self-diagnose after conducting internet searches based solely on their own criteria and conclude that they have a disease [24]. In most cases, they perceive the disease in an acute form, which increases fear and anxiety [22].

Several studies have shown an association between infectious diseases such as COVID-19, natural and social disasters, and anxiety disorders [25, 26, 27]. Individuals with high anxiety sensitivity may have beliefs that their feelings and symptoms are harmful. They then begin over-searching and worrying about their health because of what they have read on the internet [15, 19, 20, 28]. Furthermore, anxiety sensitivity has been shown as a potential risk factor for increased anxiety related to COVID-19 [27]. Individuals with severe anxiety sensitivity may find physical feelings of anxiety harmful, and as a result, try to find the cause of these feelings by searching for relevant medical information online [22].

Metacognitive beliefs have also been studied alongside PIU in relation to cyberchondria [22, 29, 30, 31]. Studies have shown that metacognitive beliefs (especially, biased thinking and beliefs about uncontrollable thoughts related to health) lead individuals to worry about their health and search the internet for medical information to reduce health anxiety [22, 30, 31]. There is likely to be a two-way relationship between PIU and cyberchondria, and metacognitive beliefs appear to play a reinforcing role between these variables. Therefore, individuals with cyberchondria can search for medical information that can increase their health anxiety, maintaining this status with metacognitive beliefs about uncontrollable health-related thoughts [22, 30].

In some countries, such as Iran, social trust in the government is low, and individuals use less official media to access important information [32, 33, 34, 35]. The low trust in the government can also cause individuals to have concerns and fears about their health. In special conditions such as the COVID-19 pandemic, there is a huge amount of misinformation on social media and on social networking sites [36, 37]. Individuals may receive misinformation from social media, which exacerbates fears of COVID-19, cyberchondria and other mental health problems. For example, in Iran, hundreds of individuals have died as a result of misinformation on social media sites about the healing properties of alcohol for the treatment of COVID-19 [38]. Since the outbreak of the disease, various studies have discussed the psychological consequences of COVID-19 [4,39,40], and have suggested different therapeutic interventions to help treat the fear and anxiety of COVID-19 [41,42]. However, in order to make effective interventions at individual and social levels, it is important to know and accurately explain the variables that affect this situation. Therefore, the present study utilized important variables identified in the research literature, and tested the following model in the general community of users of Iranian social networking sites in connection with COVID-19: (i) PIU is directly associated with cyberchondria, metacognitive beliefs, anxiety sensitivity, and fear of COVID-19; (ii) PIU is indirectly associated with fear of COVID-19 via the mediators of cyberchondria, metacognitive beliefs, and anxiety sensitivity; (iii) cyberchondria is directly associated with metacognitive beliefs, anxiety sensitivity, and fear of COVID-19; (iv) cyberchondria is indirectly associated with fear of COVID-19 via the mediators of metacognitive beliefs and anxiety sensitivity; (v) metacognitive beliefs is directly associated with anxiety sensitivity and fear of COVID-19; (vi) metacognitive beliefs is indirectly associated with fear of COVID-19 via the mediator of anxiety sensitivity; and (vii) anxiety sensitivity is directly associated with fear of COVID-19.

## 2 Methods

### 2.1 Participants, procedure, and ethics

The present study was a cross-sectional study that was conducted as a survey using an online questionnaire among the Iranian online community active on social networking sites between April and May 2020. On a page with the survey link, the text explained the objectives of the research. Individuals were informed that their participation was optional, that they had the right to leave the study at any time, and that all their data would be anonymous and confidential. Informed consent was obtained from all participants. A total of 701 social media users (including Telegram, Facebook and Twitter) clicked on the survey link, of which data from 50 participants was incomplete. Consequently, 651 survey responses were included in the final analysis. All procedures were carried out in compliance with the Helsinki Declaration. The study procedures were compliant with the Helsinki Declaration. The University Ethics Committee approved the study protocol with the reference number of IR.QUMS.REC.1399.260. All participants read and signed the online informed consent.

### 2.2 Measures

#### 2.2.1 Metacognition Questionnaire-30 (MCQ-30)

The MCQ-30 is a self-report scale comprising 30 items developed by Wells and Cartwright-Hatton [43], and includes five subscales (need to control thoughts, uncontrollability/danger, cognitive self-consciousness, positive beliefs, and cognitive confidence). Items of the MCQ-30 are rated on a four-point Likert scale (1 = *I don't agree* to 4 = *I strongly agree*). The Iranian version of the MCQ-30 has shown acceptable psychometric properties [44]. Internal consistency (Cronbach's alpha) of the MCQ-30 in the present study was 0.90.

#### 2.2.2 Anxiety Sensitivity Questionnaire (ASQ)

The ASQ is a 16-item self-report measure developed by Reiss, Peterson, Gorsky, and McNally [45] and responses are made on a five-point Likert scale (0 = *very low* to 4 = *very high*). Higher scores of the ASQ indicate more experience in fear of anxiety symptoms. The score range is between 0 and 64. The structure of the scale consists of three factors; physical concerns (eight items), cognitive concerns (four items), and social concerns (four items). The psychometric properties of the Persian language version showed promising results [46]. Internal consistency (Cronbach's alpha) of the ASQ in the present study was 0.91.

#### 2.2.3 Cyberchondria Severity Scale-Short Form (CSS-12)

The CSS-12 is a self-report scale developed by McElroy et al. [47] to assess cyberchondria behaviors. The CSS-12 has 12 items that are rated on a five-point Likert type scale from 1 (*never*) to 5 (*always*). The psychometric properties of the Persian CSS-12 have been reported to be favorable [48]. Internal consistency (Cronbach's alpha) of the CSS-12 in the present study was 0.90.

#### 2.2.4 Fear of COVID-19 scale (FCV–19S)

The FCV-19S is a self-reported scale of seven items developed by Ahorsu et al. [5] to assess fear of COVID-19. Participants' scores are rated on a five-point Likert scale from 1 (*strongly disagree*) to 5 (*strongly agree*), with a total score calculated by summing all items, and ranging from 7 to 35. Higher scores indicate greater fear of COVID-19. The internal consistency of the FCV-19S (Cronbach's alpha) in the present study was 0.87.

#### 2.2.5 Generalized Problematic Internet Use Scale (GPIUS)

The GPIUS is a self-report 29-item scale developed by Caplan [49] to assess the extent and severity of internet addiction and to assess the overall potential harms of internet addiction. GPIUS includes seven subscales (interpersonal control, withdrawal, compulsivity excess time, negative outcomes, social benefit, and mood alteration), which is scored on a five-point Likert scale from 5 (*strongly disagree*) to 5 (*strongly agree*). The validity and reliability of this scale has been confirmed among Iranian participants [50]. Internal consistency (Cronbach's alpha) of the GPIUS in the present study was 0.93.

### 2.3 Statistical analysis

For statistical analysis of this study, means and standard deviations were used as descriptive statistics. After examining the normality of the data through the Shapiro Wilk test, Pearson correlation coefficients were used to investigate the relationship between the research variables. Structural equation modeling (SEM) was used to evaluate the proposed model using full information maximum likelihood estimation via IBM SPSS AMOS 24. Model fit of the proposed model was assessed using the following recommendations: (i) Tucker-Lewis index (TLI) and comparative fit index (CFI) > 0.9; (ii) standardized root mean square residual (SRMR) and root mean square error of approximation (RMSEA) < 0.08; and (iii) non-significant chi-square [51, 52, 53]. However, the chi-square is very sensitive to a large sample size (e.g., over 200) [54]. Therefore, the data fit with the proposed model is decided using CFI, TLI, RMSEA, and SRMR. Moreover, the direct and indirect effects were evaluated using the bias-corrected bootstrapped confidence intervals (CIs) with 2,000 repetitions and a 95% confidence interval [55].

## 3 Results

Participants' demographic information is presented in Table 1. In brief, the sample (mean age = 33.53 years; SD = 10.81; age range = 13–73 years) had more females (62.5%) and more than one-third of them were single (46.5%). More than one-third of the participants had a bachelor's degree (37.8%), and more than one-third of them had Turkish ethnicity (39.3%). In the information on the history of using social networking sites, most participants used the Telegram platform (45.3%) and the maximum access time to these social networking sites was 15 times or more per day (30.1%). One-third of the sample reported that they spent 4 h daily on these social media sites (31.8%).

**Table 1:** Main participants’ characteristics (n = 651).

Sociodemographic, social media use history	n	%
Age		
13–20	62	9.5
21–40	446	68.6
41–60	128	19.6
60+	15	2.3
Gender		
Men	245	37.6
Women	406	62.4
Marital status		
Single	302	46.4
Married	312	47.9
Divorced	37	5.7
Education level		
Diploma and under diploma	156	24.0
Associate degree	59	9.1
Bachelor	246	37.8
MA	156	24.0
P.H.D	34	5.2
Race/Ethnicity		
Turkish	256	39.3
Fars	241	37.0
Kurdish	56	8.6
Arab	13	2.0
Lor	35	5.4
Baloch	10	1.5
Turkmen	10	1.5
Others	30	4.6
Social networking sites		
Telegram	295	45.3
Instagram	161	24.9
Whatsapp	87	13.4
Facebook	8	1.2
Twitter	8	1.2
Various social networks (simultaneously)	86	13.2
Others (imo, Soroush, BeeTalk and etc)	6	.9
Times accessed social network sites per day		
1–2 times	61	9.4
3–5 times	155	23.8
6–9 times	151	23.2
10–14 times	88	13.5
15 times or more	196	30.1
Time spent on social network sites per day		
0–15 min	16	2.5
16–45 min	61	9.4
46 minutes–2 hours	165	25.3
2–4 h	202	31.0
4 h or more	207	31.8

Table 2 provides the correlation between FCV-19S, CSS-12, GPIUS, ASI, and MCQ-30. The results of the correlation matrix showed that there were positive and significant correlations between fear of COVID-19 and cyberchondria (r = 0.54, *p* < .01), problematic use of the internet (r = 0.40, *p* < 0.01), anxiety sensitivity (r = 0.53, *p* < .01) and metacognitive beliefs (r = 0.36, *p* < .01).

**Table 2:** Correlations and descriptive statistics for fear of COVID-19, cyberchondria, problematic internet use, anxiety sensitivity, and metacognitions.

	M	SD	1	2	3	4	5	6	7	8	9	10	11	12	13	14	15	16	17	18	19	20	21	22	23	24
1	18.72	5.81	1																							
2	7.54	2.84	.43∗∗	1																						
3	6.44	2.73	.33∗∗	.64∗∗	1																					
4	5.68	2.64	.53∗∗	.56∗∗	.57∗∗	1																				
5	6.58	2.60	.54∗∗	.69∗∗	.59∗∗	.70∗∗	1																			
6	26.27	9.19	.54∗∗	.85∗∗	.83∗∗	.83∗∗	.87∗∗	1																		
7	8.88	2.72	.17∗∗	.17∗∗	.20∗∗	.19∗∗	.19∗∗	.22∗∗	1																	
8	12.51	4.79	.30∗∗	.19∗∗	.18∗∗	.30∗∗	.33∗∗	.29∗∗	.31∗∗	1																
9	12.62	4.05	.23∗∗	.18∗∗	.12∗∗	.26∗∗	.28∗∗	.24∗∗	.28∗∗	.55∗∗	1															
10	9.33	3.69	.34∗∗	.26∗∗	.26∗∗	.41∗∗	.38∗∗	.38∗∗	.28∗∗	.56∗∗	.63∗∗	1														
11	8.43	3.25	.37∗∗	.29∗∗	.33∗∗	.50∗∗	.41∗∗	.45∗∗	.28∗∗	.57∗∗	.42∗∗	.67∗∗	1													
12	13.20	4.54	.32∗∗	.29∗∗	.27∗∗	.37∗∗	.41∗∗	.40∗∗	.32∗∗	.55∗∗	.41∗∗	.46∗∗	.55∗∗	1												
13	12.43	4.02	.32∗∗	.20∗∗	.17∗∗	.27∗∗	.24∗∗	.26∗∗	.26∗∗	.49∗∗	.48∗∗	.40∗∗	.42∗∗	.56∗∗	1											
14	77.43	20.10	.40∗∗	.31∗∗	.29∗∗	.45∗∗	.44∗∗	.44∗∗	.49∗∗	.81∗∗	.75∗∗	.78∗∗	.75∗∗	.77∗∗	.72∗∗	1										
15	9.69	7.29	.53∗∗	.41∗∗	.37∗∗	.47∗∗	.55∗∗	.53∗∗	.27∗∗	.46∗∗	.31∗∗	.45∗∗	.47∗∗	.41∗∗	.34∗∗	.53∗∗	1									
16	4.38	3.71	.48∗∗	.38∗∗	.35∗∗	.47∗∗	.53∗∗	.50∗∗	.23∗∗	.48∗∗	.34∗∗	.48∗∗	.50∗∗	.38∗∗	.32∗∗	.54∗∗	.79∗∗	1								
17	6.88	3.11	.30∗∗	.28∗∗	.30∗∗	.25∗∗	.36∗∗	.35∗∗	.30∗∗	.36∗∗	.34∗∗	.37∗∗	.29∗∗	.34∗∗	.31∗∗	.45∗∗	.58∗∗	.52∗∗	1							
18	20.96	12.57	.53∗∗	.42∗∗	.39∗∗	.48∗∗	.56∗∗	.54∗∗	.30∗∗	.50∗∗	.37∗∗	.50∗∗	.49∗∗	.43∗∗	.37∗∗	.58∗∗	.95∗∗	.88∗∗	.74∗∗	1						
19	12.17	4.15	.27∗∗	.21∗∗	.22∗∗	.31∗∗	.31∗∗	.31∗∗	.17∗∗	.32∗∗	.19∗∗	.31∗∗	.32∗∗	.25∗∗	.19∗∗	.34∗∗	.36∗∗	.33∗∗	.32∗∗	.39∗∗	1					
20	14.33	4.47	.40∗∗	.33∗∗	.23∗∗	.34∗∗	.41∗∗	.38∗∗	.21∗∗	.38∗∗	.37∗∗	.39∗∗	.35∗∗	.36∗∗	.37∗∗	.48∗∗	.54∗∗	.55∗∗	.46∗∗	.59∗∗	.40∗∗	1				
21	11.84	4.22	.26∗∗	.22∗∗	.16∗∗	.29∗∗	.31∗∗	.29∗∗	.22∗∗	.35∗∗	.29∗∗	.35∗∗	.37∗∗	.38∗∗	.26∗∗	.43∗∗	.40∗∗	.40∗∗	.32∗∗	.43∗∗	.38∗∗	.48∗∗	1			
22	13.20	3.84	.32∗∗	.26∗∗	.23∗∗	.33∗∗	.35∗∗	.34∗∗	.21∗∗	.38∗∗	.29∗∗	.34∗∗	.34∗∗	.31∗∗	.29∗∗	.42∗∗	.46∗∗	.48∗∗	.42∗∗	.51∗∗	.56∗∗	.66∗∗	.53∗∗	1		
23	18.72	4.167	.08∗	.18∗∗	.22∗∗	.09∗	.13∗∗	.18∗∗	.20∗∗	.10∗∗	.08∗	0.06	0.06	.09∗	.11∗∗	.13∗∗	.15∗∗	.13∗∗	.28∗∗	.20∗∗	.47∗∗	.40∗∗	.19∗∗	.50∗∗	1	
24	70.28	15.72	.36∗∗	.32∗∗	.28∗∗	.36∗∗	.40∗∗	.40∗∗	.27∗∗	.41∗∗	.33∗∗	.39∗∗	.38∗∗	.37∗∗	.32∗∗	.48∗∗	.51∗∗	.51∗∗	.48∗∗	.56∗∗	.74∗∗	.79∗∗	.69∗∗	.86∗∗	.68∗∗	1

Notes: 1- Fear of COVID-19; 2. Excessiveness; 3. Reassurance 4. Compulsion 5. Distress 6. Cyberchondria 7. Interpersonal control 8. Withdrawal 9. Excessive time 10. Compulsivity 11. Negative outcomes 12. Social benefit 13. Mood alteration 14. Problematic internet use 15. Physical concern 16. Cognitive concern 17. Social concern 18. Anxiety sensitivity 19. Positive beliefs 20. Uncontrollability and danger 21. Cognitive confidence 22. Need to control thoughts 23. Cognitive self-consciousness 24. Metacognitions.

∗*p* < 0.05; ∗∗*p* < 0.01.

**Figure 1:**
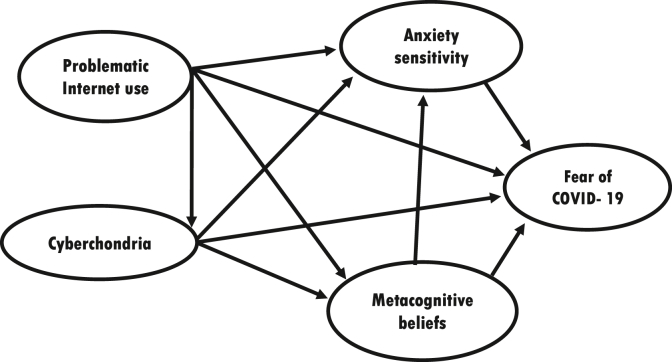
Proposed model.

The model tested by the SEM yielded an acceptable fit to the data, χ^2^ = 709.975, df = 251, RMSEA = 0.053 (90% CI = 0.048, 0.058), SRMR = 0.001, CFI = 0.948, TLI = 0.938. As shown in Figure 1, fear of COVID-19 was significantly and directly predicted by cyberchondria (standardized coefficient (β) = 0.479, *p* < .001) and anxiety sensitivity (β = 0.286, *p* < .001). However, the direct effects of metacognitive beliefs (β = 0.017, *p* = .851) and PIU (β = 0.025, *p* = .654) on fear of COVID-19 were not significant.

Table 3 shows that the indirect effects of the model shown in Figure 1 are significant. These results show that the relationship between PIU with fear of COVID-19 mediated significantly by cyberchondria, anxiety sensitivity, and metacognitive beliefs. According to the results of Table 3, problematic internet use had indirect effects on metacognitive beliefs (β = 0.144; 95% confidence interval (CI) = 0.083, 0.237), anxiety sensitivity (β = 0.425; 95% CI = 0.272, 0.439), and fear of COVID-19 (β = 0.489; 95% CI = 0.092, 0.153), and cyberchondria had indirect effects on anxiety sensitivity (β = 0.94; 95% CI = 0.045, 0.126) and fear of COVID-19 (β = 0.119; 95% CI = 0.017, 0.048), and metacognitive beliefs had an indirect effect on fear of COVID-19 (β = 0.110; 95% CI = 0.013, 0.046), and all indirect effects were significant (*p* < .001).

**Table 3:** Direct, indirect, and total standard effects.

Parameter	Total Effect (*p*-value)	Direct Effect (*p*-value)	Indirect Effect (*p*-value)	Bootstrapping SE (LLCI, ULCI)
Problematic Internet use → Cyberchondria	.585 (<0.001)	.585 (<0.001)	–	–
Problematic Internet use → Metacognitive beliefs	.640 (<0.001)	.496 (<0.001)	.144 (<0.001)	.034 (.083, .237)
Problematic Internet use → Anxiety sensitivity	.691 (<0.001)	.266 (<0.001)	.425 (<0.001)	.041 (.272, .439)
Problematic Internet use → Fear of COVID-19	.514 (<0.001)	.025 (<0.654)	.489 (<0.001)	.046 (.092, .153)
Cyberchondria → Metacognitive beliefs	.246 (<0.001)	.246 (<0.001)	–	–
Cyberchondria → Anxiety sensitivity	.400 (<0.001)	.306 (.654)	.094 (<0.001)	.023 (.045, .126)
Cyberchondria → Fear of COVID-19	.598 (<0.001)	.479 (<0.001)	.119 (<0.001)	.030 (.017, .048)
Metacognitive beliefs → Anxiety sensitivity	.384 (.026)	.384 (.851)	–	–
Metacognitive beliefs → Fear of COVID-19	.127 (<0.026)	.017 (<0.851)	.110 (<0.001)	.033 (.013, .046)
Anxiety sensitivity → Fear of COVID-19	.286 (<0.001)	.286 (<0.001)	–	–

## 4 Discussion

The present study investigated the mediating roles of anxiety sensitivity and metacognitive beliefs in the relationship between PIU and cyberchondria with fear of COVID-19 among an Iranian population. The results showed that the proposed model fitted well with the empirical data and was able to explain the role of PIU and cyberchondria by mediating anxiety sensitivity and metacognitive beliefs, in the fear of COVID-19. The direct effect of PIU on fear of COVID-19 was not meaningful, but the total and indirect paths were significant. These findings are inconsistent with the results of a study by Garcia-Priego et al. (2020) which reported a linear relationship between PIU and fear of COVID-19. However, various studies have noted the importance of using the internet during the COVID-19 pandemic period [56, 57]. Nevertheless, few studies have highlighted the importance of PIU in the mental health problems associated with COVID-19 [15]. The findings of the present study found individuals who have some psychopathological conditions (especially anxiety disorders) and problematic use of the internet may increase the fear of COVID-19.

The present research findings also showed that indirect effect of cyberchondria on fear of COVID-19 was significantly mediated by anxiety sensitivity, which is consistent with the results of research by Jungmann and Witthöft [58] and the combined role of health anxiety with cyberchondria in COVID-19 anxiety. Previous research has shown the potential roles of anxiety sensitivity in a number of anxiety disorders; for example, post-traumatic stress disorder, panic disorder, and obsessive-compulsive disorder [22]. Individuals, who use social networking sites and social media for medical purposes, usually report more anxiety and cyberchondria behaviors than normal users [27, 28, 59]. During the period of the COVID-19 pandemic, it is possible that these individuals search for information and medical news related to COVID-19 with high sensitivity to anxiety, which leads to an increase in their fear [27].

The direct effect of metacognitive beliefs on the fear of COVID-19 was non-significant. However, the total and indirect effects on fear of COVID-19 in the proposed model were significant. Until now, and to the best of the present authors' knowledge, no study has ever examined the relationship between metacognitive beliefs and fear of COVID-19. However, in a study conducted by Teovanovic et al. [57], the association of irrational beliefs and adherence to guidelines and pseudoscientific practices to protect against COVID-19 was assessed, and the results showed that irrational beliefs predicted COVID-19-related health behaviors. Therefore, the findings of the present study were somewhat inconsistent with the findings of Teovanovic et al. More specifically, the present study's results showed metacognitive barriers cannot solely predict fear of COVID-19. There may be psychological contexts or variables (including anxiety sensitivity) which can lead to fear of COVID-19 among individuals, indirectly. Beliefs about the uncontrollability of thoughts and fears related to COVID-19 may lead individuals to worry about their health and, as a result, to search the internet and social networking sites for health information and news related to COVID-19 to reduce their health anxiety. Continuation of this process will lead to the strengthening of beliefs about the uncontrollable thoughts associated with health guidelines associated with COVID-19.

Given that any research has its shortcomings, the present study is no exception and has the following limitations. First, a convenience sample of Iranian internet users was used. Therefore, the findings of this study should be considered in the context of its community, and caution should be exercised in generalizing to individuals with lower internet usage and to non-Iranians. Second, the present study is a cross-sectional survey which cannot provide evidence for causality between the variables examined. Third, all the data were self-report and are therefore subject to well-known methods biases. Finally, the present study used a general population sample rather than patients with anxiety disorders. Therefore, it is recommended that this study should be replicated among clinical groups of individuals with anxiety disorders to determine the nature of the explanatory power of this model.

## 5 Conclusion

In conclusion, the results of the present study provide sufficient evidence to support the present study's proposed model. More specifically, the model explained the role of PIU and cyberchondria directly and indirectly via anxiety sensitivity and metacognitive beliefs concerning fear of COVID-19. The results of the study can be used in clinical settings by identifying vulnerable populations at risk of biological and psychological problems and to intervene effectively.

## Declarations

### Author contribution statement

S. G. Seyed Hashemi: Conceived and designed the experiments; Wrote the paper.

S. Hosseinnezhad, Chung-Ying Lin, Amir H. Pakpour: Performed the experiments; Analyzed and interpreted the data; Wrote the paper.

S. Dini: Conceived and designed the experiments; Performed the experiments; Analyzed and interpreted the data; Wrote the paper.

Mark D. Griffiths: Analyzed and interpreted the data; Wrote the paper.

### Funding statement

This research was supported by Qazvin University of Medical Sciences.

### Competing interest statement

The authors declare no conflict of interest.

### Additional information

No additional information is available for this paper.

## References

[1] World Health Organization (2020). Coronavirus disease 2019 (COVID-19) situation report – 157. https://www.who.int/docs/default-source/coronaviruse/situation-reports/20200625-covid-19-sitrep-157.pdf?sfvrsn=423f4a82_2.

[2] Enitan S.S., Ibeh I.N., Oluremi A.S., Olayanju A.O., Itodo G.E. (2020). The 2019 novel coronavirus outbreak: current crises, controversies and global strategies to prevent a pandemic. Int. J. Path. Res..

[3] Nicola M., Alsafi Z., Sohrabi C., Kerwan A., Al-Jabir A., Iosifidis C., Agha M., Agha R. (2020). The socio-economic implications of the coronavirus pandemic (COVID-19): a review. Int. J. Surg..

[4] Mamun M.A., Griffiths M.D. (2020). First COVID-19 suicide case in Bangladesh due to fear of COVID-19 and xenophobia: possible suicide prevention strategies. Asian. J. Psychiatr..

[5] Ahorsu D.K., Lin C.Y., Imani V., Saffari M., Griffiths M.D., Pakpour A.H. (2020). The fear of COVID-19 scale: development and initial validation. Int. J. Ment. Health Addiction.

[6] Chang K.C., Hou W.L., Pakpour A.H., Lin C.Y., Griffiths M.D. (2020). Psychometric testing of three COVID-19-related scales among people with mental illness. Int. J. Ment. Health Addiction.

[7] Lin C.Y., Broström A., Griffiths M.D., Pakpour A.H. (2020). Investigating mediated effects of fear of COVID-19 and COVID-19 misunderstanding in the association between problematic social media use and distress/insomnia, Internet. Interv.

[8] Chen I.H., Chen C.Y., Pakpour A.H., Griffiths M.D., Lin C.Y. (2020). Internet-related behaviors and psychological distress among schoolchildren during COVID-19 school suspension. J. Am. Acad. Child Adolesc. Psychiatry.

[9] Pakpour A.H., Griffiths M.D., Chang K.C., Chen Y.P., Kuo Y.J., Lin C.Y. (2020). Assessing the fear of COVID-19 among different populations: a response to Ransing et al. Brain Behav. Immun..

[10] Ahorsu D.K., Imani V., Lin C.Y., Timpka T., Broström A., Updegraff J.A., Årestedt K., Griffiths M.D., Pakpour A.H. (2020). Associations between fear of COVID-19, mental health, and preventive behaviours across pregnant women and husbands: an actor-partner interdependence modeling. Int. J. Ment. Health Addiction.

[11] Pakpour A.H., Griffiths M.D., Lin C.Y. (2020). Assessing the psychological response to the COVID-19: a response to Bitan et al. “Fear of COVID-19 scale: psychometric characteristics, reliability and validity in the Israeli population”. Psychiatr. Res..

[12] Pakpour A.H., Griffiths M.D., Lin C.Y. (2020). Assessing psychological response to the COVID-19: the fear of COVID-19 scale and the COVID stress scales. Int. J. Ment. Health Addiction.

[13] Fu L., Wang B., Yuan T., Chen X., Ao Y., Fitzpatrick T., Li P., Zhou Y., Lin Y., Duan Q., Luo G. (2020). Clinical characteristics of coronavirus disease 2019 (COVID-19) in China: a systematic review and meta-analysis. J. Infect..

[14] Bhuiyan A.I., Sakib N., Pakpour A.H., Griffiths M.D., Mamun M.A. (2020). COVID-19-related suicides in Bangladesh due to lockdown and economic factors: case study evidence from media reports. Int. J. Ment. Health Addiction.

[15] Király O., Potenza M.N., Stein D.J., King D.L., Hodgins D.C., Saunders J.B., Griffiths M.D., Gjoneska B., Billieux J., Brand M., Abbott M.W. (2020). Preventing problematic internet use during the COVID-19 pandemic: consensus guidance. Compr. Psychiatr..

[16] White R., Horvitz E. (2009). Cyberchondria: studies of the escalation of medical concerns in Web search. ACM Trans. Inf. Syst..

[17] Harris P. (2008). “Cyberchondriacs” on the Rise? Those Who Go Online for Healthcare Information Continues to Increase. http://www.harrisinteractive.com/vault/HIHarris-Poll-Cyberchondriacs-2010-08-04.pdf.

[18] Office for National Statistics (2017). Internet access - households and individuals. https://www.ons.gov.uk/peoplepopulationandcommunity/householdcharacteristics/homeinternetandsocialmediausage/bulletins/internetaccesshouseholdsandindividuals/2017.

[19] Gao J., Zheng P., Jia Y., Chen H., Mao Y., Chen S., Wang Y., Fu H., Dai J. (2020). Mental health problems and social media exposure during COVID-19 outbreak. PLos One.

[20] Garcia-Priego B.A., Triana-Romero A., Pinto-Galvez S.M., Duran-Ramos C., Salas-Nolasco O., Reyes M.M., de la Medina A.R., Troche J.M. (2020). Anxiety, depression, attitudes, and internet addiction during the initial phase of the 2019 coronavirus disease (COVID-19) epidemic: a cross-sectional study in Mexico, medRxiv. https://www.medrxiv.org/content/10.1101/2020.05.10.20095844v1.

[21] van den Heuvel O.A., Veale D., Stein D.J. (2014). Hypochondriasis: considerations for ICD-11. Br. J. Psychiatry.

[22] Vismara M., Caricasole V., Starcevic V., Cinosi E., Dell'Osso B., Martinotti G., Fineberg N.A. (2020). Is cyberchondria a new transdiagnostic digital compulsive syndrome? A systematic review of the evidence. Compr. Psychiatr..

[23] McMullan R.D., Berle D., Arnáez S., Starcevic V. (2019). The relationships between health anxiety, online health information seeking, and cyberchondria: systematic review and meta-analysis. J. Affect. Disord..

[24] Starcevic V., Berle D. (2013). Cyberchondria: towards a better understanding of excessive health-related internet use. Expert Rev. Neurother..

[25] Witthauer C., Gloster A.T., Meyer A.H., Goodwin R.D., Lieb R. (2014). Comorbidity of infectious diseases and anxiety disorders in adults and its association with quality of life: a community study. Front. Publ. Health.

[26] Lee E., Lee H. (2019). Disaster awareness and coping: impact on stress, anxiety, and depression. Psychiatr. Care.

[27] Elhai J.D., Yang H., McKay D., Asmundson G.J. (2020). COVID-19 anxiety symptoms associated with problematic smartphone use severity in Chinese adults. J. Affect. Disord..

[28] Norr A.M., Capron D.W., Schmidt N.B. (2014). Medical information seeking: impact on risk for anxiety psychopathology. J. Behav. Ther. Exp. Psychiatr..

[29] Fergus T.A., Spada M.M. (2017). Cyberchondria: examining relations with problematic internet use and metacognitive beliefs. Clin. Psychol. Psychother..

[30] Fergus T.A., Spada M.M. (2018). Moving toward a metacognitive conceptualization of cyberchondria: examining the contribution of metacognitive beliefs, beliefs about rituals, and stop signals. J. Anxiety Disord..

[31] Brown R.J., Skelly N., Chew-Graham C.A. (2019). Online health research and health anxiety: a systematic review and conceptual integration. Clin. Psychol. Sci. Pract..

[32] Wang Y., Mark G. (2016). News trustworthiness and verification in China: the tension of dual media channels. Clin. Hemorheol. and Microcirc..

[33] Lu Z., Jiang Y., Lu C., Naaman M., Wigdor D. (2020 Apr 21). The government's dividend: complex perceptions of social media misinformation in China. Proceedings of the 2020 CHI Conference on Human Factors in Computing Systems.

[34] Seddighi H. (2020). Trust in humanitarian aid from the earthquake in 2017 to COVID-19 in Iran: a policy analysis, Disaster, Med, Public, Health. Prep..

[35] Lin C.Y., Imani V., Majd N.R., Ghasemi Z., Griffiths M.D., Hamilton K., Hagger M.S., Pakpour A.H. (2020). Using an integrated social cognition model to predict COVID-19 preventive behaviours. Br. J. Health Psychol..

[36] Mattiuzzi C., Lippi G. (2020). Which lessons shall we learn from the 2019 novel coronavirus outbreak?. Ann. Transl. Med..

[37] Morley J., Taddeo M., Floridi L. (2019). Google health and the NHS: overcoming the trust deficit. Lancet Digit. Health..

[38] Pleasance C. (2020). 600 people have been killed and 3,000 left in hospital in Iran after they drank neat alcohol in the mistaken belief it cures coronavirus. https://www.dailymail.co.uk/news/article-8196535/600-people-died-Iran-drinking-neat-alcohol-cure-coronavirus.html.

[39] Shevlin M., McBride O., Murphy J., Miller J.G., Hartman T.K., Levita L., Mason L., Martinez A.P., McKay R., Stocks T.V., Bennett K.M. (2020). Anxiety, depression, traumatic stress, and COVID-19 related anxiety in the UK general population during the COVID-19 pandemic. http://psyarxiv.com/hb6nq.

[40] Holmes E.A., O'Connor R.C., Perry V.H., Tracey I., Wessely S., Arseneault L., Ballard C., Christensen H., Silver R.C., Everall I., Ford T. (2020). Multidisciplinary research priorities for the COVID-19 pandemic: a call for action for mental health science. Lancet Psychiatry.

[41] Duan L., Zhu G. (2020). Psychological interventions for people affected by the COVID-19 epidemic. Lancet Psychiatry.

[42] De Sousa A., Mohandas E., Javed A. (2020). Psychological interventions during COVID-19: challenges for low and middle income countries. Asian. J. Psychiatr..

[43] Wells A., Cartwright-Hatton S. (2004). A short form of the metacognitions questionnaire: properties of the MCQ-30. Behav. Res. Ther..

[44] Shirinzadeh Dastgiri S., Gudarzi M.A., Ghanizadeh A., Taghavi S.M. (2008). Comparison of metacognitive and responsibility beliefs in patients with obsessive-compulsive disorder, generalized anxiety disorder and normal individuals, Iran. J. Psychiatr. Clin. Psychol..

[45] Reiss S., Peterson R.A., Gursky D.M., McNally R.J. (1986). Anxiety sensitivity, anxiety frequency and the prediction of fearfulness. Behav. Res. Ther..

[46] Birami M., Akbari E., Ghasempoor A., Azimi Z. (2012). An Investigation of anxiety sensitivity, meta worry and components of emotion regulation in students with and without social anxiety. Clin. Psychol. Stud..

[47] McElroy E., Kearney M., Touhey J., Evans J., Cooke Y., Shevlin M. (2019). The CSS-12: development and validation of a short-form version of the cyberchondria severity scale. Cyberpsychol., Behav. Soc. Netw..

[48] Dini S., Hosseinnezhad S., Hosseinzadeh Khanmiri B., Seyed Hashemi S.G. (2020). Internal consistency and confirmatory factor analysis of the short-form version of cyberchondria severity scale among social networking sites users. Second National Congress of New Findings in Psychology and Counseling.

[49] Caplan S.E. (2002). Problematic internet use and psychosocial well-being: development of a theory-based cognitive-behavioral measurement instrument, Comput. Human. Beyond Behav..

[50] Alavi S.S., Jannatifard F., Maracy M., Rezapour H. (2009). The psychometric properties generalized pathological internet use scale (GPIUS) in internet users students of Isfahan Universities. J. Know. Res. Appl. Psychol..

[51] Schweizer K. (2010). Some guidelines concerning the modeling of traits and abilities in test construction. Eur. J. Psychol. Assess..

[52] Lin C.Y., Cheng A.S.K., Imani V., Saffari M., Ohayon M.M., Pakpour A.H. (2020). Advanced psychometric testing on a clinical screening tool to evaluate insomnia: sleep condition indicator. Sleep Biol. Rhythm.

[53] Lin C.Y., Imani V., Griffiths M.D., Pakpour A.H. (2020). Psychometric properties of the Persian Generalized Trust Scale: confirmatory factor analysis and Rasch models and relationship with quality of life, happiness, and depression. Int. J. Ment. Health Addiction.

[54] Wu T.H., Chang C.C., Chen C.Y., Wang J.D., Lin C.Y. (2015). Further psychometric evaluation of the Self-Stigma Scale-Short: measurement invariance across mental illness and gender. PloS One.

[55] Lin C.Y., Tsai M.C. (2016). Effects of family context on adolescents’ psychological problems: moderated by pubertal timing, and mediated by self-esteem and interpersonal relationships. Appl. Res. Qual. Life..

[56] Hernández-García I., Giménez-Júlvez T. (2020). Assessment of health information about COVID-19 prevention on the internet: infodemiological study. JMIR. Public. Health. Surveill..

[57] Li C., Chen L.J., Chen X., Zhang M., Pang C.P., Chen H. (2020). Retrospective analysis of the possibility of predicting the COVID-19 outbreak from internet searches and social media data, China, 2020. Euro Surveill..

[58] Jungmann S.M., Witthöft M. (2020). Health anxiety, cyberchondria, and coping in the current COVID-19 pandemic: which factors are related to coronavirus anxiety?. J. Anxiety Disord..

[59] El Sherif R., Pluye P., Thoër C., Rodriguez C. (2018). Reducing negative outcomes of online consumer health information: qualitative interpretive study with clinicians, librarians, and consumers. J. Med. Internet Res..

