# Corrigendum to: “The mediating effect of the cyberchondria and anxiety sensitivity in the association between problematic internet use, metacognition beliefs, and fear of COVID-19 among Iranian online population” [Heliyon 6 (10), (October 2020), Article e05135]

**DOI:** 10.1016/j.heliyon.2022.e09335

**Published:** 2022-04-29

**Authors:** Seyed Ghasem Seyed Hashemi, Shalaleh Hosseinnezhad, Solmaz Dini, Mark D. Griffiths, Chung-Ying Lin, Amir H. Pakpour

**Affiliations:** a Department of Psychology, Faculty of Psychology and Educational Sciences, Azarbaijan Shahid Madani University, Tabriz, Iran; b Department of Psychology, Urmia Branch, Islamic Azad University, Urmia, Iran; c Department of Psychology, Faculty of Psychology and Educational Sciences, Bonab Branch, Payame Noor University, Bonab, Iran; d International Gaming Research Unit, Psychology Department, Nottingham Trent University, Nottingham, UK; e Department of Rehabilitation Sciences, Hong Kong Polytechnic University, Hung Hom, Hong Kong; f Institute of Allied Health Sciences, National Cheng Kung University Hospital, College of Medicine, National Cheng Kung University, Tainan, Taiwan; g Social Determinants of Health Research Center, Research Institute for Prevention of Non-Communicable Diseases, Qazvin University of Medical Sciences, Qazvin, Iran

In the original published version of this article, the article was incorrectly stated as having been submitted to the journal in January 2020. This has now been corrected to August 2020. The publisher apologizes for this error. In addition, the original published version of this article stated the incorrect institutional review board reference number as IR.QUMS.REC.1399.260 in the Methods section. This has now been corrected to IR.QUMS.REC.1398.375. The authors apologise for this mistake. Both the HTML and PDF versions of the article have been updated to correct the error.

